# Young Bedouin-Arab Men’s Ego and Pride: Do Traditional Masculinity and Positive Attitudes Toward Polygyny Shape Responses to a Wife’s Refusal?

**DOI:** 10.3390/bs14111081

**Published:** 2024-11-12

**Authors:** Avi Besser, Virgil Zeigler-Hill, Nuzha Allassad Alhuzail

**Affiliations:** 1 Department of Communication Disorders, Hadassah Academic College, Jerusalem 9101001, Israel; 2 Department of Psychology, Oakland University, Rochester, MI 48309, USA; zeiglerh@oakland.edu; 3 School of Social Work, Sapir Academic College, “Shaar HaNegev” Educational Campus, Ashkelon Beach 7915600, Israel

**Keywords:** traditional masculinity, Bedouin-Arab, young men, polygynous marriage

## Abstract

Cultural traditions in Israel’s Bedouin-Arab community encourage and permit men to take up to four wives, a practice supported by Islamic teachings. Despite legal prohibitions against polygyny in Israel, such marriages remain common and have profound effects on women, children, and broader society. This study explores how traditional notions of masculinity and positive attitudes toward polygyny influence young Bedouin men’s reactions to a hypothetical scenario in which their wife refuses to accept a polygynous marriage as a threat to men’s ego and pride. The sample consisted of 459 young, unmarried Israeli Bedouin-Arab men, aged 16 to 25, a demographic frequently under family pressure to marry, making the issue of marriage highly relevant. We hypothesized that traditional masculinity and favorable attitudes toward polygyny would be positively associated with heightened perceptions of ego and pride threats in response to a wife’s refusal. Participants were randomly assigned to imagine either their wife refusing a polygynous arrangement (*n* = 228) or accepting a polygynous arrangement (*n* = 231) and then reporting their anticipated emotional responses. The results showed that traditional masculinity was linked to positive attitudes toward polygyny, and both were significantly associated with increased perceptions of ego and pride threats in the refusal scenario but not in the acceptance scenario. The findings suggest that men with strong masculine identities and favorable views of polygyny are especially vulnerable to feeling threatened by a wife’s refusal. This supports social construction theory and underscores the role of cultural norms in shaping emotional responses. The discussion highlights the psychological impacts of polygyny in patriarchal societies and calls for culturally sensitive interventions that challenge traditional norms while fostering psychological resilience and gender equality.

## 1. Introduction

Polygamous marriage, where a man marries multiple wives (polygyny) or a woman marries multiple husbands (polyandry), is particularly prevalent worldwide in its polygynous form [1]. Despite legal prohibitions in many societies, polygamous marriages continue to persist across various cultures and religions [2]. Global rates of polygamy vary significantly, ranging from 4% to 54% depending on geographical location [3,4]. Although polygamous marriages have generally declined, adapted forms are increasingly seen in developing nations [2].

In Israel, polygyny is particularly recognized among Bedouin-Arab communities in the Negev region, though official statistics on its prevalence are sparse [5]. Typically, these marriages involve one man and two or more women, often leading to decreased marital satisfaction and instances of abuse [6]. Under Islamic Sharia law, men may marry up to four wives provided they can support them equally, contributing to the 20% to 36% polygyny rate among the Negev’s Bedouin-Arab population [7,8]. Recent estimates suggest that approximately 18.5% to 21.6% of Bedouins are engaged in polygynous marriages [9,10]. Although Sharia permits polygyny, Israeli law criminalizes it. Nonetheless, polygyny is on the rise, especially among younger, educated men [10,11], reflecting the underlying patriarchal dynamics of a Bedouin-Arab society in transition [12].

Several factors contribute to the persistence of polygyny, including the disadvantaged status of Bedouin-Arab women within this patriarchal system [13] and the attribution of masculine traits to men married to more than one woman [14].

Legal prohibitions are often unenforced, allowing men to marry according to Sharia law without official registration. Psychological issues among co-wives, such as depressive symptoms, low self-esteem, and reduced life satisfaction, are well documented [10,15]. Dynamics among co-wives frequently foster jealousy and conflict, further complicating the unequal distribution of marital and familial responsibilities [16]. Furthermore, when monogamous marriages transition to polygynous ones, senior wives may experience trauma, a phenomenon known as “First Wife Syndrome” [17,18]. Daughters can also be affected by their father’s remarriage, reporting feelings of loss and reduced paternal presence [13].

While much research has focused on the benefits men perceive in polygynous families and the challenges faced by wives and children [19,20], there has been little examination of individual differences among Muslim or Arab men who prefer polygyny, particularly Bedouin men. A recent study of young single Bedouin-Arab women found strong opposition to polygyny, which they perceived as a threat to their relationships [21]. This underscores the critical gap in understanding the attitudes of young Bedouin men toward polygyny and their cultural and psychological motivations. This study seeks to explore how masculinity and positive attitudes toward polygyny influence young Bedouin men’s responses to a hypothetical refusal from their wives to engage in polygyny, particularly in terms of threats to their ego and pride.

The current study examines how “young men” navigate the decision to accept or reject cultural expectations surrounding polygyny and the subsequent impact on their well-being as they transition into adulthood. This topic is especially relevant as Bedouin society undergoes changes that require men to navigate a complex social landscape, balancing traditional values with modern demands that challenge the phenomenon of polygyny. Cultural expectations and family pressures significantly shape Bedouin masculinity, impacting both personal choices and community perceptions [14]. Men are encouraged to marry more than one woman to address the phenomenon of singlehood among Bedouin women, and having multiple wives is considered a mark of status in the traditional masculinity index. Bedouin culture actively promotes polygyny, as reflected in the proverb “A good man is one with two or three wives” [14].

A study examining attitudes toward polygyny among high school students and older individuals in a Bedouin-Arab town in the Negev revealed significant gender differences. The research found that women were more likely to view polygyny as a source of familial conflict and broader societal issues, such as poverty and increased educational disparities. In contrast, men generally supported the practice [22].

This study is grounded in social construction theory, which posits that masculinity is shaped by social relations and contextual factors [23,24]. Through this lens, we aim to understand how societal expectations, norms, and collective identities influence Bedouin men’s perceptions of polygyny and their emotional responses to potential refusals. Social construction theory provides a framework for analyzing the influence of traditional masculinity on attitudes toward polygyny while also revealing the cultural narratives that sustain these beliefs. This perspective enhances our understanding of how masculinity is expressed within the Bedouin context and adapts to changing social dynamics. Moreover, it allows for a critical examination of cultural frameworks and gender roles, contributing to a more nuanced understanding of masculinity. While traditional Bedouin masculinity often emphasizes responsibility for family stability [25], hegemonic masculinities in other societies may focus on dominance and patriarchal power [24,26]. Exploring these contrasts offer valuable insights into how cultural frameworks influence gender roles while also highlighting the unique dimensions of Bedouin masculinity.

Research by Allassad Alhuzail and Nabbary (2023) found that young Bedouin men feel a stronger sense of belonging to their tribe than to their village, underscoring the tribal structure’s influence on Bedouin masculinity. This sense of belonging highlights the importance of tribal identity in framing societal roles, social expectations, and personal identity, which are all deeply embedded in the societal fabric and daily lives of Bedouin communities [27].

Despite limited research on individual differences, little is known about how these differences may explain young Bedouin-Arab men’s preferences for polygyny or how they manage stress related to potential threats by entering such marriages. This study will examine traditional masculinity and positive attitudes toward polygyny. Racialized Muslim masculinities often position Muslim men as defenders of patriarchal norms, intertwining hegemonic masculinity with compulsory heterosexuality deeply embedded within Muslim family dynamics [28,29,30,31].

Furthermore, issues such as gender-based violence and sexual deviance perpetuate negative portrayals of racialized Muslim masculinities [32,33]. In Israel’s Bedouin community, masculinity reflects both traditional patriarchal structures and modern influences amid sociocultural challenges [34]. Although religious teachings advocate equality, significant gender inequality persists within Bedouin society [12,35], sparking political activism in response to discrimination [36,37].

Educated Bedouin men face the challenge of balancing traditional values with modern societal norms [8,38,39]. While they often support women’s social mobility, they are often hesitant to challenge traditional roles [40]. Their masculinity is tied to honor and familial reputation, reflecting collective cultural values [41,42].

**Hypothesis** **1.** *Traditional masculinity is expected to positively correlate with positive attitudes toward polygyny. Men with high traditional masculinity are likely to hold positive views of polygynous marriage.*

**Hypothesis** **2.** *Traditional masculinity is expected to be associated heightened emotional responses, particularly concerning threatened ego and pride, when faced with a woman’s hypothetical refusal to participate in a polygynous marriage. The rationale for this prediction is that masculinity, while based on biological factors, represents an ideology promoting specific traits and behaviors [43]. Achieving and maintaining masculinity requires continuous adherence to societal expectations, as it is fragile and “hard-won and easily lost” [44]. A wife’s refusal of polygynous marriage is likely to amplify the connection between traditional masculinity and negative emotional responses.*

**Figure 1:**
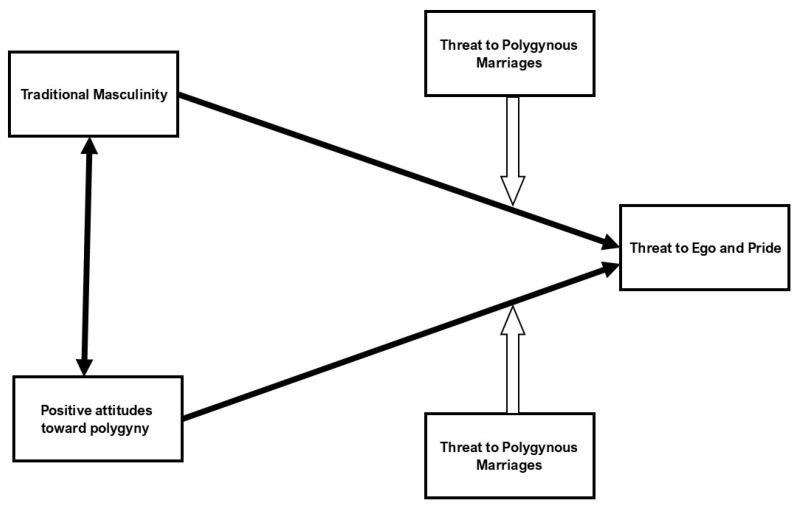
An illustration of the relationship between men’s traditional masculinity, their positive attitudes toward polygynous marriages, and the negative emotional responses related to ego and pride. It highlights how these relationships are influenced by whether a wife accepts or refuses to enter into a polygynous marriage.

**Hypothesis** **3.** *Positive attitudes toward polygyny are expected to be associated heightened emotional responses, particularly concerning threatened ego and pride, when faced with a woman’s hypothetical refusal to participate in a polygynous marriage. The rationale for this prediction is that the refusal may be particularly threatening to the ego and pride of these men because polygyny is often associated with power, control, and the fulfillment of traditional male responsibilities within their community. In contrast, men with less favorable attitudes toward polygyny may not place as much importance on it as part of their identity, and therefore they may experience less emotional turmoil when faced with refusal in this context. The heightened emotional response among men who favor polygyny may be further intensified by the cultural pressure to maintain patriarchal authority and meet societal expectations. Figure 1 presents the proposed associations between men’s traditional masculinity, their positive attitudes toward polygynous marriages, and the negative emotional responses related to ego and pride, as well as the moderating role of a wife’s acceptance or refusal to enter into a polygynous marriage.*

## 2. Materials and Methods

### 2.1. Participants

The study utilized a convenience sample of 459 unmarried and unengaged Israeli Bedouin-Arab men from the Negev region, who volunteered to participate by responding to recruitment efforts through flyers placed in public spaces and posts shared on various social media platforms. Participants completed an online survey hosted on a secure website, beginning with an online informed consent form. They then completed a demographic questionnaire and measures assessing traditional masculinity and attitudes toward polygamous marriages. Afterward, participants read the specific hypothetical scenario to which they were randomly assigned and completed a measure assessing the perception of refusal of polygynous marriage as a threat. Participation was restricted to young men between the ages of 16 and 25, as this group represents a transitional period into adulthood when many men (and women) are expected to be engaged or married due to family pressure. This makes the topic of marriage particularly relevant and emotionally significant for them. Although the study was not pre-registered, the data file is publicly accessible on the Open Science Framework (OSF) at https://osf.io/cq8gf/.

### 2.2. Procedure

This study was conducted in three phases: pre-manipulation, manipulation, and post-manipulation. In the pre-manipulation phase, participants completed questionnaires assessing their masculinity, attitudes toward polygyny, and current emotional state, as well as other questionnaires that are not relevant for the present study (e.g., personality). During the manipulation phase, participants were asked to imagine the following scenario:

“Please imagine you have been married for about three years and you have a little baby. One evening, you and your wife are sitting in the living room you say to your wife: ‘I have fallen in love with other women, and I have decided to marry her. It will be good for both of us and for our relationship.’”

Participants were then randomly assigned to read one of two hypothetical scenarios designed to evoke either the wife’s refusal of polygyny (*n* = 228) or acceptance of polygyny (*n* = 231). The scenarios diverge at this point, with the refusal scenario concluding with the following description:

“After taking a moment to consider your words, your wife responds with deep conviction, ‘Absolutely not. I believe this would harm us and our relationship. Just the thought of you taking another woman causes me immense distress. I would rather get divorced.’”

In contrast, the acceptance scenario concluded with the following description: “After taking a moment to consider your words, your wife responds with deep conviction, ‘I fully agree. I believe that taking another wife will enrich our lives and strengthen our relationship. The idea brings me a sense of peace and optimism.’”

### 2.3. Questionnaires

#### 2.3.1. Pre-Manipulation Measure

##### Traditional Masculinity

Masculinity was measured using statements *taken* from a validated measure [45]. The six statements included the following: *I consider myself as*…; *Ideally, I would like to be*…; *Traditionally, my interests would be considered as*…; *Traditionally, my attitudes and beliefs would be considered as*…; *Traditionally, my behavior would be considered as*…; *Traditionally, my outer appearance would be considered as*… (*α* = 0.94). Participants responded using a scale from 1 (*not at all masculine*) to 7 (*totally masculine*).

##### Attitudes Toward Polygamous Marriages

Attitudes were measured using statements derived from a validated measure [22]. The four statements were as follows: *Polygamy causes conflicts between couples*; *Polygamy causes conflicts between the husband’s family and the wife*; *Polygamy causes conflicts between the children of the co-wives*; *Polygamy causes social problems, such as poverty* (*α* = 0.89). Participants rated their level of agreement with each statement on a scale ranging from 1 (*do not agree at all*) to 5 (*agree very much*). Scores were reversed to measure positive attitudes.

#### 2.3.2. Post-Manipulation Measures

##### Refusal of Polygynous Marriage as a Threat

After the manipulation, participants rated the extent to which their wife’s refusal or acceptance of polygyny threatened or boosted their ego and pride using a scale from 1 (*not at all*) to 7 (*very much*). In the acceptance condition, scores were reversed to assess threat perception.

### 2.4. Ethics Statement

Participation was voluntary, and participants could withdraw at any time. Signed, informed consent was obtained, and no social security numbers or other identifying data were collected, nor were any invasive examinations conducted. This study was conducted in accordance with the Declaration of Helsinki and approved by the Ethics Committee (IRB) of Hadassah Academic College (protocol code #00551 [31 August 2024]).

### 2.5. Statistical Analysis

We first analyzed the Pearson product–moment correlation coefficients among the variables, followed by comparisons of traditional masculinity, positive attitudes toward polygyny, and post-manipulation anticipated threat to ego and pride. A hierarchical multiple regression analysis was then conducted to determine whether polygyny refusal moderated the associations that traditional masculinity and positive attitudes toward polygyny had with anticipated threat to one’s ego and pride. This was accomplished by regressing perception of anticipated threat to one’s ego and pride onto traditional masculinity, positive attitudes toward polygyny, and the polygyny refusal condition (−1 = *polygyny acceptance*, 1 = *polygyny refusal*), with all continuous predictor variables centered for the purpose of testing interactions [46]. The main effects were entered on Step 1, the two-way interactions on Step 2, and the three-way interaction on Step 3. Simple slopes tests [46] were performed to clarify the interactions, using values one standard deviation above and below the mean to represent high or low levels of the variables.

## 3. Results

### 3.1. Comparison of Refusal Conditions Based on Sociodemographic and Study Variables

Table 1 presents the sociodemographic information for the participants. The results indicate that there were no significant differences in sociodemographic characteristics between participants in the refusal and acceptance conditions. These findings show that the random assignment effectively created comparable groups regarding age, education, and other background variables. Therefore, sociodemographic factors were excluded from the final analysis to ensure clarity and conciseness.

Table 2 illustrates that participants in the refusal condition exhibited no significant differences from those in the acceptance condition regarding traditional masculinity or positive attitudes toward polygyny. This suggests that the random assignment effectively resulted in comparable levels of these factors prior to the manipulation. As expected, participants in the refusal condition reported greater responses concerning threats to their ego and pride compared to those in the acceptance condition.

### 3.2. Univariate Analyses

The correlation coefficients for the overall sample are presented in Table 3a, while those for the refusal and acceptance conditions are shown in Table 3b. Supporting Hypothesis 1, traditional masculinity was positively correlated with positive attitudes toward polygyny across the entire sample, as well as in both the refusal and acceptance conditions. In line with our hypotheses, traditional masculinity (Hypothesis 2) and positive attitudes toward polygyny (Hypothesis 3) were significantly correlated with post-manipulation threats to ego and pride for the entire sample. Furthermore, these correlations were significant in the refusal condition but not in the acceptance condition.

### 3.3. Hierarchical Moderated Multiple Regression Results

**Figure 2:**
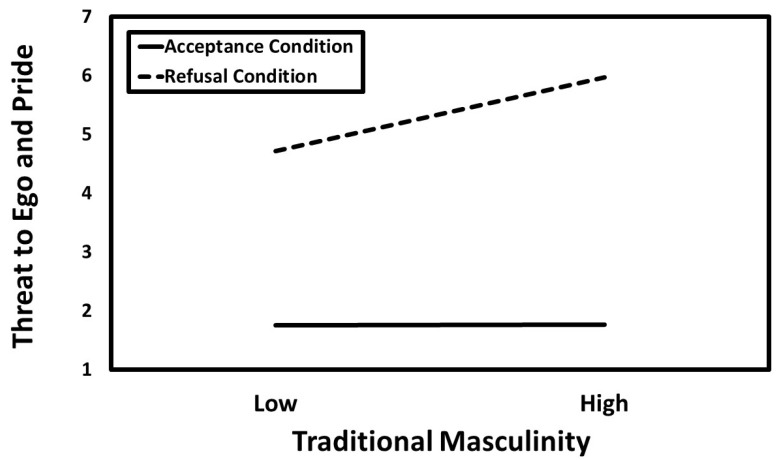
The predicted values illustrating the interaction that traditional masculinity had with the refusal condition for perceived threat to ego and pride.

**Figure 3:**
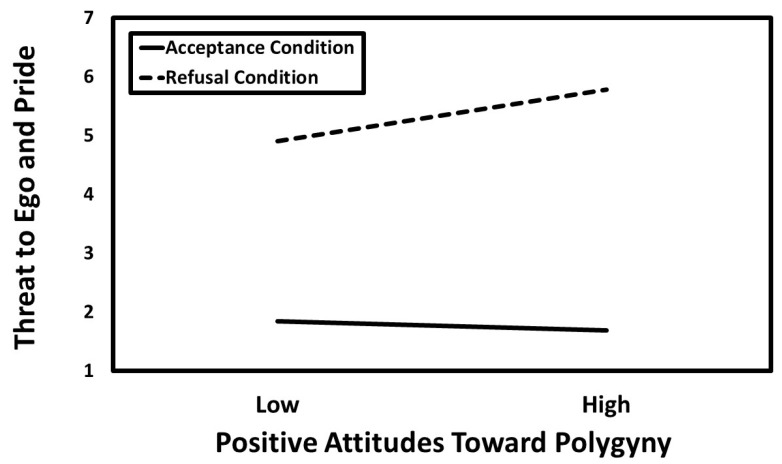
The predicted values illustrating the interactions that positive attitudes toward polygyny had with the refusal condition for perceived threat to ego and pride.

Table 4 displays the results of the hierarchical moderated multiple regression analysis. The results revealed significant positive main effects for traditional masculinity, positive attitudes toward polygyny, and refusal. Additionally, the two-way interactions between traditional masculinity and the refusal condition, as well as between positive attitudes toward polygyny and the refusal condition, were also significant. The predicted values for these interactions are presented in Figure 2 and Figure 3. Simple slopes tests indicated that the association between masculinity and perceived threat to ego and pride was significant in the refusal condition (*B* = 0.46, *SE* = 0.09, *t* = 5.20, *p* < 0.001, *CI_95%_* [0.29, 0.64]) but not in the acceptance condition (*B* = 0.00, *SE* = 0.09, *t* = 0.01, *p* = 0.993, *CI_95%_* [−0.17, 0.17]). Similarly, the association between attitudes toward polygyny and perceived threat to ego and pride was significant in the refusal condition (*B* = 0.31, *SE* = 0.08, *t* = 4.06, *p* < 0.001, *CI_95%_* [0.16, 0.46]) but not in the acceptance condition (*B* = −0.06, *SE* = 0.07, *t* = −0.80, *p* = 0.423, *CI_95%_* [−0.20, 0.08]).

**Figure 4:**
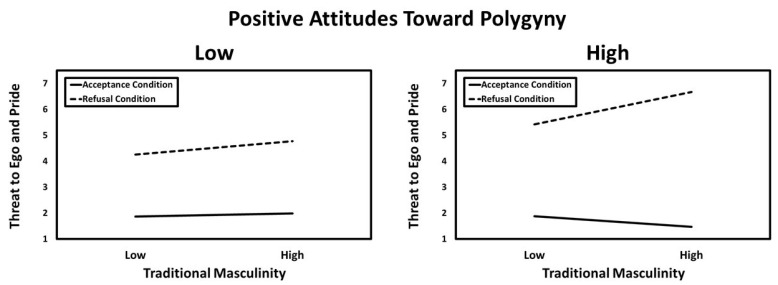
The predicted values illustrating the interaction between traditional masculinity, positive attitudes toward polygyny, and the refusal condition for perceived threat to ego and pride.

A significant three-way interaction was found among traditional masculinity, positive attitudes toward polygyny, and the refusal condition. The predicted values for this interaction are presented in Figure 4. Simple slopes tests revealed that the association between traditional masculinity and perceived threat to ego and pride was significant in the refusal condition when positive attitudes toward polygyny were high (*B* = 0.70, *SE* = 0.15, *t* = 4.64, *p* < 0.001, *CI_95%_* [0.40, 0.99]). In contrast, this association was not significant in the acceptance condition when positive attitudes toward polygyny were high (*B* = −0.19, *SE* = 0.15, *t* = −1.25, *p* = 0.213, *CI_95%_* [−0.48, 0.11]). Furthermore, the association between masculinity and perceived threat to ego and pride was not significant in the refusal condition (*B* = 0.02, *SE* = 0.03, *t* = 0.58, *p* = 0.559, *CI_95%_* [−0.05, 0.09]) or the acceptance condition (*B* = 0.00, *SE* = 0.08, *t* = 0.03, *p* = 0.977, *CI_95%_* [−0.16, 0.16]) when positive attitudes toward polygyny were low.

## 4. Discussion

This study aimed to investigate how traditional masculinity and favorable attitudes toward polygyny are associated with young Bedouin men’s responses to their wives’ potential refusal of polygynous arrangements, particularly concerning threats to their ego and pride. As noted by Zeitzen (2008), the ongoing practice of polygyny in certain cultural and religious contexts, despite legal prohibitions, underscores deeply ingrained patriarchal norms and the influence of cultural traditions on marital expectations [2]. The findings of this study offer valuable insights into the complex interplay between traditional masculinity, attitudes toward polygyny, and the psychological responses of young Bedouin-Arab men when faced with the potential refusal of polygyny from their wives.

Our findings confirm the hypotheses that traditional masculinity and positive attitudes toward polygyny are associated with heightened sensitivity to ego and pride threats when faced with a wife’s refusal. Furthermore, this study indicates that men who hold both traditional masculine values and positive attitudes toward polygyny are the most susceptible to experiencing feelings of ego and pride threat following a wife’s refusal to enter into a polygynous arrangement. This sensitivity was particularly pronounced in the refusal condition, echoing Connell and Messerschmidt’s (2005) suggestion that these cultural constructs shape emotional responses to challenges against traditional gender roles [24]. This aligns with the observation that masculinity is often constructed through familial expectations and social interactions, as evident in Bedouin culture, where there is strong encouragement for men to marry multiple wives as a response to the phenomenon of singlehood among Bedouin girls and as a counter-reaction to laws prohibiting these marriages [14].

This cultural reinforcement is encapsulated in the Bedouin proverb “A good man is one with two or three wives” [14]. Deeply rooted in Bedouin cultural values, this saying likely influences men’s perceptions of themselves and their social standing, potentially shaping their emotional responses when faced with a wife’s refusal of polygyny. By examining such cultural narratives, we can gain insight into how social construction theory illuminates the development of these beliefs and their impact on individuals’ responses to potential threats to masculine identity. The cultural pressure reflected in this proverb, coupled with the heightened sensitivity to threats concerning ego and pride observed in this study, further underscores the complex interplay between cultural norms, traditional masculinity, and men’s responses to social expectations within Bedouin communities.

It is crucial to recognize that the scenarios in our study involved distinct emotional and psychological dynamics. In the refusal condition, participants faced not only a wife’s direct rejection of polygyny but also a statement expressing emotional distress, culminating in an implicit threat of divorce. This combination likely heightened participants’ perceptions of emotional threat. In contrast, the other scenario lacked a parallel to the implicit threat of divorce, which raises the following question: were the men’s feelings of ego and pride threat driven by the refusal alone, the implied threat of divorce, or a combination of both? Addressing this complexity is essential, as it may significantly influence the interpretation of these findings. We acknowledge this nuance and stress the importance of future research to explore how varying degrees of emotional threat are experienced by young men in polygynous contexts. By understanding these nuances, we can gain deeper insight into the psychological mechanisms at work when individuals face potential refusals in traditional relationships.

It is crucial to acknowledge that polygyny often has profound and complex implications for women, particularly within a patriarchal context. While this study focuses on men’s experiences, it is important to recognize that women in polygynous marriages often face significant challenges, including a heightened risk of social and psychological distress. Research has documented the traumatic experience of “First Wife Syndrome,” where senior wives experience emotional distress and a sense of loss following their husband’s subsequent marriage [17,18]. This syndrome—often triggered by feelings of abandonment, insecurity, and diminished social standing—can have long-lasting negative effects on women’s well-being. By acknowledging ‘First Wife Syndrome’ and its potential impact on women, we recognize the complex and nuanced reality of polygyny within Bedouin communities. Furthermore, social construction theory sheds light on the often-overlooked roles women play within the dynamics of polygynous marriages. Understanding women’s perspectives and their interactions within these relationships is crucial for a comprehensive analysis of how traditional masculinity not only shapes men’s attitudes but also influences women’s experiences and expectations. Further research should explore how societal interventions aimed at promoting gender equality and addressing the harmful effects of patriarchal norms might mitigate the negative consequences of polygyny for women and promote a more just and equitable environment for all members of the community. In various studies, Al-Krenawi has investigated the impact of polygamy on not only women and children but also men, revealing that the practice can lead to negative emotional and social consequences for all parties involved. This research highlights issues such as economic strain, marital dissatisfaction, and emotional stress experienced in polygamous marriages [5,22,47,48]. Despite these negative impacts on both genders, the phenomenon persists and even continues to grow. It is possible that the community’s efforts to preserve its culture and traditions include maintaining polygamy as a means of cultural and communal continuity.

Furthermore, the observed findings resonate with Daoud et al. (2014), who emphasized that cultural norms and masculine identities considerably influence conflict resolution and emotional expression within Bedouin-Arab communities [10]. The emphasis on family honor and reputation is deeply tied to men’s roles, reflecting collective societal values [41,42]. Although young Bedouin men are becoming more educated and are navigating modern societal challenges, they grapple with the pressures of conforming to traditional masculinity [8,14,38], which often leads to internal conflict and stress [27]. This stress may be heightened when confronted with potential spousal refusal regarding polygynous arrangements. By adopting a social construction framework, we gain a deeper understanding of how internalized cultural values are associated with these men’s responses to challenges to their masculinity, including potential rejection by their wives. This perspective allows us to see traditional masculinity not merely as a set of behaviors but as a socially constructed identity that men actively negotiate and perform in their daily lives.

This study also highlights the role of patriarchal dynamics in shaping gender relations and maintaining traditional practices like polygyny, which are noted to significantly impact familial and psychological well-being [10,15]. Despite becoming more educated, young Bedouin men still often feel the pressure to adhere to traditional masculine ideals that prioritize control and dominance. This internalization can lead to heightened emotional sensitivity under stress, particularly in scenarios involving potential spousal rejection regarding polygynous arrangements.

These tendencies among young Bedouin men gain significance considering that, while polygyny is widespread and represents 20–36% of marriages within the Negev Bedouin-Arab population [7,10], 31.4% of the current sample reported their families as being polygynous. Findings from a recent study involving young, unmarried Israeli Bedouin-Arab women from the same region and age group as the men in the current study reveal a strong resistance to polygynous marriages: 80.4% of their sample perceived their husbands taking multiple wives as a significant threat to their relationships [21]. In contrast, the men in the current sample were generally supportive of polygynous marriages, with 70% scoring at or above the midpoint of the scale measuring positive attitudes toward polygyny. Additionally, 41% expressed the strongest possible level of support for polygyny, scoring a “5” on a scale ranging from 1 to 5. This disparity in attitudes may reflect the diverging socialization experiences of men and women within this cultural context, highlighting the need for an analytical framework that views gender dynamics as socially constructed rather than as fixed identities. Such an understanding underscores how societal norms shape the expectations placed on men and women within polygynous settings.

Additionally, it is essential to note that Bedouin society is undergoing transformative processes, rapidly evolving from a collective and patriarchal structure [40,49]. This transformation is characterized by increased access for women to higher education and greater participation in the labor market, resulting in a significant rise in entrepreneurial initiatives led by Bedouin women [50]. Overall, both the observed differences in gender attitudes toward polygyny and these evolving dynamics may heighten potential interpersonal and intrapersonal conflicts. These conflicts may be further exacerbated by the increased investment by women to integrate into higher education and the workforce, coupled with less investment by young men, leading to a disparity within the Bedouin family. The adherence to traditional masculinity and the power position granted to men by the patriarchal structure likely sustains the positive attitudes of men toward polygyny, explaining its continued existence despite the changes and transitions that Bedouin society is experiencing.

Despite these contributions, this study has several limitations. Firstly, the use of a convenience sample from a specific demographic group—unmarried young Bedouin men from Israel, particularly from the Negev region—limits the generalizability of the findings to other groups or regions. Secondly, the reliance on hypothetical scenarios might not fully capture the real-life complexities and emotions involved in marital dynamics. Additionally, self-reported measures might be subject to biases such as social desirability, potentially affecting the accuracy of the participants’ representations of their attitudes.

Future research should investigate these dynamics across diverse sociocultural settings and include different demographic groups, such as married men and women, to provide a broader understanding of the psychological aspects of polygynous marriages. Longitudinal studies could examine how traditional masculinity and attitudes toward polygyny evolve over time and impact marital satisfaction and mental health. Qualitative approaches could offer deeper insights into personal experiences and societal influences on attitudes toward polygyny. Furthermore, identifying the factors that shape these attitudes and perceptions could help to facilitate effective interventions. Programs aimed at fostering gender equality and supporting emotional resilience should consider incorporating these findings to promote healthier relationship dynamics within Bedouin communities and similar settings. By applying a social constructionist lens to future research, scholars can illuminate the complex interplay between gender, culture, and individual behaviors, helping to challenge harmful stereotypes and develop more equitable frameworks for understanding relationships in these contexts. This approach can enrich our understanding of the changing nature of gender roles, especially as societies navigate the tension between modernity and tradition.

## 5. Conclusions

To the best of our knowledge, this study is the first to investigate how individual differences that may affect how young Bedouin men cope with the stress and negative reactions related to the prospect of entering a polygynous marriage. Our findings have significant implications for understanding polygynous relationships within Bedouin-Arab culture, offering vital insights into how traditional masculinity and attitudes toward polygynous unions shape the psychological responses of young Bedouin-Arab men when faced with potential rejection from their spouses. By delving into the cultural and emotional dynamics at play, we shed light on the intricate interplay between established patriarchal norms and the challenges brought about by modernization.

While acknowledging this study’s limitations, such as the specific demographics of the sample and the hypothetical nature of the scenarios presented, our results underscore the need for inclusive research that encompasses diverse demographic groups and real-life experiences. Future research should expand its focus to include married individuals and various cultural contexts, employing qualitative and longitudinal methodologies to deepen our understanding of these dynamics. Furthermore, initiatives aimed at promoting gender equality and strengthening emotional resilience will be crucial in fostering healthier relationship patterns. By addressing these factors, we can help to achieve a harmonious balance between traditional values and modern realities, ultimately enhancing the emotional well-being and social cohesion of Bedouin communities and similar cultural settings.

## Figures and Tables

**Table 1:** Sociodemographic variables.

	Total Sample (*N* = 459)	Acceptance Condition (*n* = 231)	Refusal Condition (*n* = 228)	Statistics	
				***t*=**	***p*=**
Mean Age	18.86 (2.45)	19.03 (2.05)	18.69 (2.80)	1.46	0.15
Years of Formal Education	12.22 (1.26)	12.32 (1.11)	12.13 (1.40)	1.60	0.11
				***χ* ^2^**	***p*=**
*Student Status*				0.095	0.78
Current Student	57.7%	58.4%	57%		
Not a Student	42.3%	41.6%	43%		
*Socioeconomic Status*				5.71	0.22
Very Good	16.6%	14.7%	18.4%		
Good	32.5%	29.0%	36.0%		
Average	39.7%	43.3%	36.0%		
Bad	8.7%	10.4%	7.0%		
Very Bad	2.6%	2.6%	2.6%		
*Religious Beliefs*				0.37	0.83
Religious	29.4%	28.6%	30.3%		
Traditional	63.8%	64.1%	63.6%		
Secular	6.8%	7.4%	6.1%		
*Family Structure*				0.009	0.93
Monogamous	68.6%	68.8%	68.4%		
Polygynous	31.4%	31.2%	31.6%		

**Table 2:** Comparisons of the refusal and acceptance conditions.

	Acceptance Condition (*n* = 231)		Refusal Condition (*n* = 228)		*t*	*CI_95%_*
	*M*	*SD*	*M*	*SD*		
Traditional Masculinity	6.28	1.27	6.20	1.46	0.64	−0.17, 0.33
Positive Attitudes Toward Polygyny	3.59	1.42	3.68	1.45	−0.65	−0.35, 0.18
Perceived Threat to Ego and Pride	1.78	1.38	5.36	1.86	−23.47 ***	−3.88, −3.28

*** *p* < 0.001.

**Table 3:** (a) The intercorrelations and descriptives for the entire sample (*N* = 459). (b) The intercorrelations and descriptive statistics for the acceptance (*n* = 231) and refusal conditions (*n* = 228).

(a)	1	2	3
1. Traditional Masculinity	---		
2. Positive Attitudes Toward Polygyny	0.31 ***	---	
3. Perceived Threat to Ego and Pride	0.15 ***	0.15 ***	---
			
*M*	6.24	3.63	3.56
*SD*	1.37	1.44	2.42
*Skewness*	−2.16	−0.56	0.33
*Kurtosis*	4.31	−1.12	−1.49
**(b)**	**1**	**2**	**3**
1. Traditional Masculinity	---	0.41 ***	0.44 ***
2. Positive Attitudes Toward Polygyny	0.20 **	---	0.38 ***
3. Perceived Threat to Ego and Pride	−0.03	−0.06	---
			
*M_Acceptance Condition_*	6.28	3.59	1.78
*SD_Acceptance Condition_*	1.27	1.42	1.38
*Skewness_Acceptance Condition_*	−2.23	−0.50	1.66
*Kurtosis_Acceptance Condition_*	5.12	−1.17	1.60
*M_Refusal Condition_*	6.20	3.68	5.36
*SD_Refusal Condition_*	1.46	1.45	1.86
*Skewness_Refusal Condition_*	−2.09	−0.63	−0.54
*Kurtosis_Refusal Condition_*	3.67	−1.05	−1.33

The values below the diagonal are taken from participants in the acceptance condition, whereas the values above the diagonal are taken from participants in the refusal condition. ** *p* < 0.01; *** *p* < 0.001.

**Table 4:** The results of the hierarchical moderated multiple regression analysis for perceived threat to ego and pride.

	*B*	*SE*	*t*	*p*	*CI_95%_*
*Step 1*					
Traditional Masculinity	0.26	0.06	4.50	<0.001	0.14, 0.37
Positive Attitudes Toward Polygyny	0.14	0.05	2.65	=0.008	0.04, 0.25
Refusal Condition	1.79	0.07	24.41	<0.001	1.65, 1.94
*Step 2*					
Traditional Masculinity	0.23	0.07	3.35	<0.001	0.095, 0.37
Positive Attitudes Toward Polygyny	0.13	0.05	2.40	=0.017	0.02, 0.23
Refusal Condition	1.79	0.07	25.25	<0.001	1.65, 1.93
+ Traditional Masculinity × Positive Attitudes Toward Polygyny	0.02	0.03	0.58	=0.56	−0.05, 0.09
+ Traditional Masculinity × Refusal Condition	0.23	0.06	4.18	<0.001	0.12, 0.34
+ Positive Attitudes Toward Polygyny × Refusal Condition	0.18	0.05	3.51	<0.001	0.08, 0.28
*Step 3*					
Traditional Masculinity	0.24	0.07	3.43	<0.001	0.10, 0.37
Positive Attitudes Toward Polygyny	0.13	0.05	2.48	=0.013	0.03, 0.23
Refusal Condition	1.74	0.07	23.75	<0.001	1.60, 1.89
Traditional Masculinity × Positive Attitudes Toward Polygyny	0.01	0.03	0.42	=0.67	−0.05, 0.08
Traditional Masculinity × Refusal Condition	0.33	0.07	4.76	<0.001	0.19, 0.46
Positive Attitudes Toward Polygyny × Refusal Condition	0.18	0.05	3.42	<0.001	0.08, 0.28
+ Traditional Masculinity × Positive Attitudes Toward Polygyny × Refusal Condition	0.08	0.03	2.33	=0.02	0.01, 0.15

Note: Step 1: *R*^2^ = 0.582, *F*(3, 455) = 211.09, *p* < 0.001; Step 2: *R*^2^ = 0.618, *F*(6, 452) = 121.74, *p* < 0.001; ΔStep 2: Δ*R*^2^ = 0.036, Δ*F*(3, 452) = 14.12, *p* < 0.001; Step 3: *R*^2^ = 0.622, *F*(7, 451) = 106.15, *p* < 0.001; ΔStep 3: Δ*R*^2^ = 0.005, Δ*F*(1, 451) = 5.450, *p* = 0.02.

## Data Availability

The data presented in this study are available on request from the corresponding authors.
